# Survival of *Bifidobacterium longum* LMG 13197 microencapsulated in Vegetal or Vegetal-inulin matrix in simulated gastrointestinal fluids and yoghurt

**DOI:** 10.1186/s40064-016-3010-y

**Published:** 2016-08-12

**Authors:** Andreal Chioma Amakiri, Mapitsi Silvester Thantsha

**Affiliations:** 1 Department of Microbiology and Plant Pathology, University of Pretoria, Lynwood Road, Pretoria, 0002 South Africa; 2 Department of Microbiology and Plant Pathology, Faculty of Natural and Agricultural Sciences, University of Pretoria, New Agricultural Sciences Building, Lunnon Road, Pretoria, 0002 South Africa

**Keywords:** Lipid excipients, Microencapsulation, Probiotics, Vegetal, Inulin, Simulated gastrointestinal fluids

## Abstract

**Background:**

Vegetal BM 297 ATO is a food grade lipid based material extracted from vegetables, and certified for human consumption. In this study, *Bifidobacterium longum* LMG 13197 was encapsulated in Vegetal BM 297 ATO-inulin by freeze drying, followed by evaluation of its survival in simulated gastrointestinal fluids and yoghurt. Furthermore, the effect of incorporation of such microparticles on physico-chemical properties of yoghurt was examined. Unencapsulated and encapsulated *B. longum* cells were exposed to simulated gastrointestinal fluids for 6 h and yoghurt at 4 °C for 6 weeks, and then evaluated for viability using plate counts.

**Results:**

By the end of exposure to simulated gastrointestinal fluids, encapsulated cells were >5 log units higher than their unencapsulated counterparts. Furthermore, their levels in yoghurt remained above 10^6^ cfu mL^−1^ until the end of 6 weeks of storage while unencapsulated levels were at this level up to 5 weeks. There were no significant differences in pH values of yoghurts containing encapsulated cells throughout the storage (p > 0.05). However, significant differences in the lightness and yellowness of these yoghurts were recorded, although the total colour change was negligible.

**Conclusions:**

Vegetal-inulin encapsulation protected probiotics in gastrointestinal fluids and yoghurt with negligible effects to its appearance, thus can be used for fortification of yoghurt with probiotics.

## Background

Probiotics have been described as “live microorganisms which when administered in adequate amounts confer a health benefit on the host” (FAO/WHO 2002). Over the years, consumers have become aware of the benefits of ingesting fermented foods which contain probiotics. Yogurt is one of the oldest fermented milk products and the most common dairy products consumed around the world (Shah 2000). The purpose for its production and consumption is to improve health and reduce the occurrence of gastrointestinal diseases (Roberfroid 2007). The increasing popularity of yogurt has led food industries and various researchers to continuously investigate how best to add value to yoghurt products to attract health-conscious consumers (Allgeyer et al. 2010). More recently, yoghurt has been fortified with live microorganisms called probiotics, mainly lactobacilli and bifidobacteria. However, viability of probiotics and the universal quality of many probiotic-containing products is still a major issue, as the number of viable probiotics and especially bifidobacteria decline over time in dairy foods during storage and subsequently during gastrointestinal transit (Sultana et al. 2000). Poor survival and viability of bifidobacteria in yoghurt results in counts lower than 10^6^–10^7^ cfu g^−1^, which is the recommended daily intake needed to confer health benefits (Doleyres and Lacroix 2005). Probiotic survival in fermented products is affected by a range of factors including pH, dissolved oxygen, storage temperature and post-acidification (Shah 2000).

In order to protect probiotic cultures during processing, storage in food products and during gastrointestinal transit, innovative technologies such as microencapsulation, as well as the addition of prebiotics have been explored (Fritzen-Freire et al. 2012). Studies have shown that microencapsulation improves probiotic survival during transit in the human gastrointestinal transit, during manufacturing and storage in yoghurt (Kailasapathy 2006; Thantsha et al. 2009). Prebiotics have received increasing attention because of their beneficial health effects as well as their ability to improve food quality (Capela et al. 2006). In food technology, prebiotics such as inulin have been used to improve texture and mouthfeel, as stabilizers, fat replacers, and flavour enhancers in food products such as yoghurt (Pimentel et al. 2013). Inulin has also been used as ingredients in functional foods as well as dietary supplements, because they are regarded safe for consumption (Kelly 2009).Products containing both probiotic and prebiotic components are generically termed synbiotics (Kalliomäki 2009). These products affords the food industries with a strategy to both provide physical barriers to protect the probiotics from detrimental conditions, as well as give suitable nutrients that will selectively enhance proliferation of the probiotics (Doleyres and Lacroix 2005; Thantsha et al. 2009).

During the process of microencapsulation, coating materials such as starch, gellan gum, alginate and k-carrageenan have been mostly utilized (Burgain et al. 2011). However, the use of food grade lipid coating materials for preparation of synbiotic products suitable for incorporation into foods is yet to be fully investigated. Vegetal BM 297 ATO is a glyceryl distearate produced from vegetables with melting temperatures between 53 and 58 °C. Vegetal can be used as a dietary supplement and thus is certified safe for human consumption (Gattefossé SAS, technical and material data sheets 2010). The aim of this study was to assess the effect of encapsulation with Vegetal BM 297 ATO in conjunction with inulin on the survival of *Bifidobacterium longum* LMG 13197in simulated gastrointestinal fluids, yoghurt and the resultant effect of the microparticles on the physico-chemical properties of yoghurt.

## Methods

### Reagents and bacterial cultures

Biogapress Vegetal BM 297 ATO was obtained in powdered form from Gattefossé SAS (France). *B. longum* LMG 13197 cultures were obtained in freeze-dried form from BCCM/LMG Culture collection (Belgium), revived according to the manufacturer’s specifications and then kept as 20 % glycerol stocks in de Man, Rogosa, Sharpe (MRS) broth (Sigma Aldrich, South Africa) at −70 °C. Inulin (purity: 95 %), polyvinyl alcohol (PVA) 87–89 % partially hydrolysed (Mw: 13,000–23,000 Da), lactose monohydrate (purity: 99 %) were obtained from Sigma Aldrich, South Africa, while dichloromethane (DCM) (analytical grade, purity: 99 %) was obtained from Sigma Aldrich Laborchemikalien, Seelze.

### Encapsulation of bacteria

One millilitre of overnight *B. longum* LMG 13197 culture was subcultured into three 250 mL flasks containing 100 mL MRS-cys-HCl broth, and incubated overnight in a shaking incubator (250 rpm) at 37 °C. Cells were then harvested by centrifugation, using an Eppendorf centrifuge 5804R (at 4 °C) at 20,800×*g* for 15 min. The pelleted cells (approximately 3.14 × 10^8^ cfu mL^−1^) were washed once with Ringer’s solution and kept at 4 °C for 5 min before encapsulation. The first emulsion was prepared by suspending the bacterial pellet into 1 mL of (2 % w/v) inulin. The bacteria-inulin mixture was then added to 1 mL of (2 % w/v) poly-vinyl-alcohol (PVA). The resulting suspension was subsequently added to 10 mL of dichloromethane (DCM) containing (10 % w/v) Vegetal BM 297 ATO. This emulsion was homogenized at 8000 rpm for 5 min using a Silverson, L4R, NIMR homogenizer and left to stand at 25 °C. The second emulsion was prepared by mixing 15 mL of (2 % w/v) PVA and 5 mL of (5 % w/v) lactose. The first emulsion was mixed into the second emulsion and homogenised at 8000 rpm for 5 min using a Silverson, L4R, NIMR homogenizer (Stewart and Brierley Pty Ltd., South Africa). The stable emulsion was left to stand in the fume hood for 5 h for DCM evaporation. After evaporation of DCM, the sample was frozen at −20 °C overnight. This was followed by freeze drying using a Virtis bench top, SLC, freeze dryer for 3 days at −75 °C. The freeze dryer was set at a condenser temperature and vacuum pressure of −60 °C and 0.26 millitor, respectively.

The same protocol was used to prepare Vegetal BM 297 ATO microparticles encapsulating *B. longum* LMG 13197 without inulin, except bacterial pellet was re-suspended in 1 mL of deionised water before mixing with 1 mL of (2 % w/v) PVA. The unencapsulated cells was prepared by re-suspending *B. longum* cells (approximately 3.14 × 10^8^ cfu mL^−1^) into 25 mL of sterile ¼ strength Ringer’s solution and fast-frozen in liquid nitrogen. The fast-frozen cells were then frozen at −70 °C for 1 h, and then freeze dried as was done for encapsulated cells. All the freeze dried samples were stored in tightly sealed sterile Schott bottles at 4 °C for further analysis within 10 h.

### Survival of bacteria in simulated gastrointestinal fluids (SGIF)

Simulated gastric fluid (SGF) was prepared according to Lian et al. (2003). Briefly, pepsin (P7000, 1:10,000, ICN, Sigma Aldrich, South Africa) (3 g L^−1^) was suspended in sterile NaCl solution (0.5 % w/v). The pH of the solution was adjusted to pH 2.0 with 12 M HCl, and then filter sterilized through a 0.22 μm filter membrane (Pall Corporation, USA). Simulated intestinal fluid (SIF) was prepared according to US Pharmacopoeial (2005). Briefly, 6.8 g of monobasic potassium phosphate (Sigma, St. Louis, MO, USA) was dissolved in 250 mL of distilled water, followed by addition of 77 mL of 0.2 M NaOH and 500 mL of distilled water. The solution was vortexed for 30 min and then 10 g of pancreatin (P-1500, Sigma, St. Louis, MO, USA) was added and mixed. The solution was adjusted to pH 6.8 with 0.2 M NaOH or 0.2 M HCl. The total volume of the solution was made up to 1000 mL, followed by filtration through a 0.45 µm filter membrane to remove particulate material, and then filter sterilized through a 0.22 µm filter membrane.

One gram of unencapsulated and encapsulated samples was then dispersed into separate test tubes containing 9 mL of SGF (pH 2.0). The tubes were vortexed for 30 s and incubated at 37 °C in a shaker incubator (Lasec, LM-575R) at 50 rpm for 2 h. One millilitre subsamples were withdrawn from the tubes at 30 min intervals for 2 h after vortexing of tubes containing the unencapsulated cells and gentle pipetting of the encapsulated samples. Bacteria in the subsamples were then enumerated using plate count assay (PCA). Bacteria remaining in SGF after withdrawal of the 2 h subsample were pelleted by centrifugation using a LabnetPrism™ Microcentrifuge at 7,267*×g* for 5 min. The pellets were resuspended in 9 mL of SIF (pH 6.8) and incubated as before. One millilitre subsamples were taken at 2 h intervals until 6 h for bacterial enumeration. After removal of each subsample, sterile SGF or SIF equal to the subsample withdrawn was added to the tube to maintain the concentration of sample.

### Survival of encapsulated *B. longum* LMG 13197 in yoghurt

Two hundred and fifty millilitres of skimmed milk was supplemented with 3 % w/v of non fat dry powdered milk in three flasks. The milk mix was homogenised by thoroughly swirling the flasks and then pasteurised at 72 °C for 30 min. It was then cooled to 42 °C, inoculated (6 % w/v, ca 1 × 10^6^ cfu mL^−1^) with lactic culture containing *Streptococcus thermophilus* and *Lactobacillus delbrueckii* subsp *bulgaricus* and incubated at 45 °C until a pH of 4.53 was attained. Yoghurt was pasteurized at 72 °C for 30 min to kill the starter cultures as previously done by Sun and Griffiths (2000), before addition of bifidobacteria. One millilitre of yoghurt samples were pour plated onto M-17 and MRS-cys-HCl agar plates to confirm absence of viable *S*. *thermophilus* and *L*. *bulgaricus*, respectively, prior to addition of bifidobacteria. Then 30 mL of the fermented yoghurt was poured into 50 mL sterile containers and aseptically mixed with 1 g of either unencapsulated or encapsulated cells and stored at 4 °C for 6 weeks. Viability of *Bifidobacterium* cells in stored yoghurt was determined weekly using PCA. The ‘0 day’ analysis was carried out after overnight cold storage of samples.

### Physico-chemical analysis of yoghurt

The pH and colour of stored yoghurts were measured weekly in triplicates. The pH was measured using a Crison Basic 20 pH meter (Denver instruments, USA). The colour was analysed using a Minolta Chroma Meter CR-400 (Konica Minolta, Osaka, Japan) calorimeter. The total colour difference ($\Delta {\text{E}}^{ *}$) between the unencapsulated and encapsulated samples was calculated as previously done by Fritzen-Freire et al. (2012) using the equation:

$$\Delta {\text{E}}^{ *} = [(\Delta {\text{L}}^{*} )^{2} + \, (\Delta {\text{a}}^{*} )^{2} + (\Delta {\text{b}}^{*} )^{2} ]^{1/2}$$

where ${\text{L}}^{*}$ represents variations from black to white, ${\text{a}}^{*}$ represents variations from red + to green − and ${\text{b}}^{*}$ represents variations from yellow + to blue −.

### Enumeration of *B*. *longum* cells

The subsamples withdrawn from SGF, SIF and yoghurt were serially diluted in ¼ strength Ringer’s solution. The samples withdrawn from yoghurt were first resuspended into 9 mL of DCM and vortexed for 30 s to disrupt the lipid matrix and release the bacteria before performing a serial dilution. Then aliquots of 100 μL of each dilution were pour-plated in triplicates onto MRS agar plates supplemented with 0.05 % cys-HCl. The plates were incubated at 37 °C for 72 h in anaerobic jars with Anaerocult A gaspaks and Anaerocult C test strips to indicate anaerobic conditions in the jar.

### Statistical analysis

A completely randomized design was used in this study and data was analysed using a one way analysis of variance (ANOVA). Mean values and standard deviations were calculated from the data obtained from three independent trials, the difference between the means was calculated using Least Significant Difference (LSD) and a p-value less than 0.05 was considered to be statistically significant. Analysis was performed using Statistica version 6.0 (StatsoftInc, Tulsa, USA).

## Results

### Survival of *B. longum* LMG 13197 in simulated gastrointestinal fluids

**Fig. 1:**
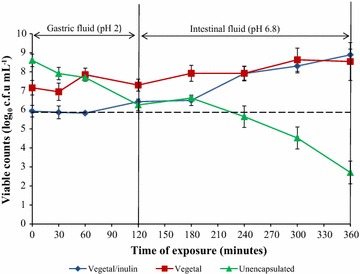
Survival of unencapsulated and encapsulated *B*. *longum* LMG 13197 after exposure to simulated gastrointestinal fluids over 6 h. Each *point* represents the average of triplicate counts from three independent trials and *error bars* are standard deviations of three replicates. The *dotted line* represents the minimum recommended viability level for probiotics

A reduction of 2.34 log_10_ cfu mL^−1^ in viable counts of unencapsulated bacteria was recorded after their exposure to SGF (Fig. 1). Vegetal encapsulated bacteria showed an initial decrease of viable counts from 7.16 to 6.95 log_10_ cfu mL^−1^ after the first 30 min of exposure to SGF (Fig. 1). This was followed by an increase in the number of cells to 7.30 log_10_ cfu mL^−1^ after 2 h. Similarly, Vegetal-inulin encapsulated bacteria showed a decrease from an initial count of 5.93 to 5.87 log_10_ cfu mL^−1^ within the first 30 min of exposure to SGF, followed by an increase in cells to 6.43 log_10_ cfu mL^−1^ after 2 h (Fig. 1). The numbers of cells released from Vegetal and Vegetal-inulin matrix at the end of exposure to SGF were 0.90 and 0.60 log_10_ cfu mL^−1^, respectively.

Upon subsequent exposure to SIF, the numbers of the unencapsulated bacteria continued to decrease, with total bacterial cell loss of 5.89 log units after 6 h (Fig. 1). The numbers of viable cells released immediately following exposure in SIF were 0.62 and 0.07 log units for Vegetal and Vegetal-inulin matrixes, respectively. Both Vegetal and Vegetal-inulin matrixes showed continuous release of cells above 10^6^ log_10_ cfu mL^−1^ in SIF, with no significant difference (p > 0.05) at the end of 6 h. The numbers of Vegetal and Vegetal-inulin encapsulated bacteria in SIF showed increases of 1.24 and 2.46 log units, respectively at the end of exposure.

### Survival of *B. longum* LMG 13197 in yoghurt and its effects on the pH and colour of yoghurt during storage

**Fig. 2:**
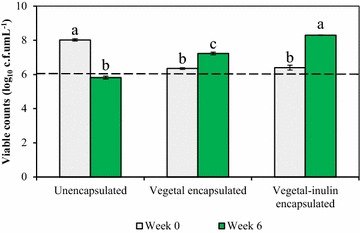
Survival of unencapsulated and encapsulated *B*. *longum* LMG 13197 in yoghurt at 4 °C for 6 weeks. Each *bar* represents the average of triplicate counts from three independent trials and *error bars* are standard deviations of three replicates. The *dotted line* represents the minimum recommended viability level for probiotics. *Different letters* denotes significant differences in viable counts between the samples

Unencapsulated cells showed 2.70 log unit reduction in viability during storage in yoghurt whereas encapsulated cells showed increases of 0.9 and 1.9 log units for Vegetal and Vegetal-inulin encapsulated cells, respectively (Fig. 2). Viable counts for bacteria encapsulated by both matrices were lower than those of unencapsulated bacteria after overnight storage, and were also less than their counts recorded after 6 weeks of storage in yoghurt. However, their levels in both occasions were above 6 log cfu, the minimum recommended level of viability for probiotics to confer the beneficial effects in consumers. Interestingly, levels of these cells were higher after 6 weeks than those of unencapsulated cells, whose levels decreased below the minimum recommended value. At the end of storage, viable numbers of bacteria encapsulated by both matrices were significantly higher (p < 0.05) than levels of unencapsulated cells. Furthermore, viability was significantly higher (p < 0.05) for Vegetal-inulin encapsulated bacteria than those encapsulated in Vegetal alone.

The pH of yoghurt containing unencapsulated bacteria decreased by 0.21, with significant differences (p < 0.05) observed from the fourth to sixth week of storage (Table 1). Conversely, the pH of yoghurts containing Vegetal and Vegetal-inulin encapsulated bacteria decreased by 0.05 and 0.06 units, respectively by the sixth week. The reduction of pH was less than what was observed with unencapsulated cells, partially explaining why viability of the cells was not affected as shown earlier (Fig. 2).

**Table 1:** Changes in pH of yoghurt samples containing unencapsulated and Vegetal encapsulated *B. longum* LMG 13197 (with and without inulin) stored at 4 °C for 6 weeks

Yoghurt samples	Storage time (weeks)						
	0	1	2	3	4	5	6
Containing unencapsulated cells	4.51 ± 0.01^c^	4.51 ± 0.02^c^	4.50 ± 0.01^c^	4.50 ± 0.01^c^	4.49 ± 0.01^c^	4.40 ± 0.10^b^	4.30 ± 0.01^a^
Containing Vegetal encapsulated cells	4.50 ± 0.01^c^	4.50 ± 0.01^c^	4.50 ± 0.01^c^	4.49 ± 0.01^bc^	4.48 ± 0.02^b^	4.45 ± 0.01^a^	4.45 ± 0.01^a^
Containing Vegetal-inulin encapsulated cells	4.52 ± 0.01^e^	4.51 ± 0.01^de^	4.51 ± 0.01^cd^	4.49 ± 0.01^bc^	4.49 ± 0.01^b^	4.46 ± 0.01^a^	4.46 ± 0.01^a^

^a^Reported values are means of triplicate readings from three independent trials. Values with different letters within the same row differ significantly (p < 0.05)

There were significant differences (p < 0.05) in colour attributes (${\text{L}}^{*}$, ${\text{a}}^{*}$ and ${\text{b}}^{*}$) within each of the yoghurt samples containing unencapsulated and encapsulated cells throughout 6 weeks of storage (Table 2). Significant increases (p < 0.05) were observed in ${\text{L}}^{*}$ and ${\text{b}}^{*}$ values throughout storage. The presence of inulin had a significant effect (p < 0.05) on the lightness (${\text{L}}^{*}$) and yellowness (${\text{b}}^{*}$) of yoghurt. A reduction in ${\text{a}}^{*}$ values with significant differences (p < 0.05) within the samples was observed throughout 6 weeks of storage.

**Table 2:** Changes in the colour of yoghurt samples containing *B. longum* LMG 13197 cells during storage at 4 °C for 6 weeks

Storage time (weeks)	Yoghurt samples											
	Colour attributes											
	Containing unencapsulated cells				Containing Vegetal encapsulated cells				Containing Vegetal-inulin encapsulated cells			
	${\text{L}}^{*}$	${\text{a}}^{*}$	${\text{b}}^{*}$	$\Delta {\text{E}}^{ *}$	${\text{L}}^{*}$	${\text{a}}^{*}$	${\text{b}}^{*}$	$\Delta {\text{E}}^{ *}$	${\text{L}}^{*}$	${\text{a}}^{*}$	${\text{b}}^{*}$	$\Delta {\text{E}}^{ *}$
0	65.82 ± 0.14^a^	−1.68 ± 1.10^c^	−0.44 ± 0.02^a^	–	63.75 ± 2.01^a^	−1.30 ± 0.03^d^	−1.02 ± 0.36^a^	0.37	77.96 ± 1.68^b^	−2.04 ± 0.18^c^	3.04 ± 0.95^b^	2.97
1	78.91 ± 0.51^b^	−2.19 ± 0.02^bc^	3.73 ± 0.24^b^		78.93 ± 1.13^b^	−2.28 ± 0.18^c^	4.02 ± 0.77^b^		68.74 ± 1.06^a^	−1.11 ± 0.11^d^	−0.18 ± 0.20^a^	
2	81.54 ± 0.90^c^	−2.42 ± 0.12^ab^	5.63 ± 0.64^cd^		80.14 ± 2.16^b^	−2.59 ± 0.31^b^	4.31 ± 0.65^b^		78.30 ± 0.96^b^	−2.13 ± 0.17^c^	3.31 ± 0.46^b^	
3	82.46 ± 0.24^d^	−2.81 ± 0.06^ab^	5.91 ± 0.18^d^		86.47 ± 0.01^d^	−3.11 ± 0.08^a^	8.63 ± 0.07^e^		86.20 ± 0.13^d^	−3.18 ± 0.04^a^	8.22 ± 0.13^d^	
4	82.20 ± 0.24^cd^	−2.24 ± 0.03^bc^	5.37 ± 0.20^c^		83.77 ± 0.09^c^	−2.42 ± 0.03^bc^	6.48 ± 0.10^c^		84.18 ± 0.32^c^	−2.43 ± 0.03^b^	6.43 ± 0.13^c^	
5	85.14 ± 0.26^e^	−3.04 ± 0.02^a^	8.33 ± 0.01^f^		83.19 ± 0.26^c^	−3.14 ± 0.13^a^	6.95 ± 0.37^cd^		86.22 ± 0.10^d^	−3.20 ± 0.06^a^	8.82 ± 0.10^d^	
6	84.51 ± 0.15^e^	−3.12 ± 0.04^a^	7.37 ± 0.14^e^		84.88 ± 0.05^cd^	−3.11 ± 0.08^a^	7.42 ± 0.17^d^		87.21 ± 0.08^d^	−3.14 ± 0.01^a^	8.61 ± 0.10^d^	

^a^Reported values are means of triplicate readings from three independent trials. Values with different letters within the same column differ significantly (p < 0.05)

## Discussion

The decrease of unencapsulated cells during exposure to SGF after 2 h has also been reported by researchers elsewhere. Hansen et al. (2002), showed a 3–4 log decrease in *B. longum* Bb46 after 2 h while De Castro-Cislaghi et al. (2012) reported a 1.51 log decrease in unencapsulated *B. lactis* Bb12 at pH 2. In comparison to the unencapsulated cells, encapsulation preserved viability of probiotic cells. The number of cells released from Vegetal matrix in SGF was higher than that of Vegetal-inulin, suggesting that addition of inulin improved the effectiveness of Vegetal to protect bifidobacteria during exposure to SGF. Therefore, encapsulation with Vegetal-inulin performed better at protecting the cells from gastric acidity.

In agreement with previous report by Thantsha et al. (2009), there was a continuous reduction in viable numbers of unencapsulated *B. longum* Bb46 cells in SIF. On the other hand, during exposure to SIF, cells encapsulated in Vegetal and Vegetal-inulin matrices were gradually released to numbers recommended for the provision of health benefits. The increase in numbers of viable cells for both encapsulated matrices indicated that in the SIF (pH 6.8), the matrices were able to spontaneously disintegrate to release the cells. A gradual release of the viable cells is much more desirable than burst release, as studies have reported that burst release leads to higher cell delivery, which can affect the ability of the matrix to maintain long term controlled release of cells (Huang and Brazel 2001). In support of our findings, Okuro et al. (2013) demonstrated that lipid microcapsules with prebiotics were disintegrated at pH 6.5, leading to the release of encapsulated bacteria. Although both matrices protected and released the cells in SIF, this study suggested that Vegetal-inulin matrices released higher numbers of viable cells. Therefore, Vegetal-inulin has more potential to efficiently protect the cells in the upper gastrointestinal tract and then release them in sufficient numbers for colonization in the lower gastrointestinal tract.

Since bifidobacteria are sensitive to low pH (Sun and Griffiths 2000), continuous exposure of the unencapsulated cells to the acidic environment of yoghurt would cause a reduction in their numbers. Our results have shown that effective release of bacteria from Vegetal matrix occurs at pH of 6.8, close to neutral. Therefore, it would be expected that the matrix would not spontaneously release most of the cells into the acidic environment of yoghurt (pH 4.5). Hence, the high numbers of viable cells released after dissolution of the matrices at the end of storage period were anticipated.

Our previous research reported encapsulation efficiencies of 88 and 82 % for Vegetal and Vegetal-inulin matrices, respectively (Amakiri et al. 2015). The actual amount of viable cells encapsulated within the Vegetal and Vegetal-inulin matrices were 7.48 and 6.97 log cfu mL^−1^, respectively. Therefore, viable counts obtained during week 0 represent viability of 85 and 92 % of the bacteria encapsulated within these matrices, respectively. These data thus indicated sufficient protection and release of *B*. *longum* cells from both matrices. Worth noting are higher levels of bacterial viability obtained for both matrices after 6 weeks, which corresponds to viability of >90 % of the encapsulated bacterial cells. Whereas an increase in numbers of viable cells was observed for encapsulated bacteria, a 68 % reduction in viability of the unencapsulated cells was observed. The high numbers of viability for encapsulated bacteria can be attributed to firstly, the ability of the encapsulating matrices to physically retain the bifidobacteria cells within, thereby protect the cells from detrimental factors such as yoghurt acidity and exposure to oxygen. Encapsulation in Vegetal-inulin matrix provided better protection for the cells, possibly because inulin provided extra solids, which have been reported to improve probiotic protection (Capela et al. 2006). The presence of inulin thus afforded the bacteria additional nutrients which enhanced their growth subsequent to their release, thereby providing more suitable growth conditions than was available for bacteria released from Vegetal matrix without inulin. Boeni and Pourahmad (2012) reported that the addition of 2 % inulin improved viability of *Lactobacillus casei* and *L. acidophilus* for 3 weeks. Similarly, Akalin et al. (2004) reported higher counts of *B. longum* in yoghurts containing prebiotics. Secondly, the higher numbers of viable cells at 6 weeks could also be due to enhanced dissolution of the matrices subsequent to suspension of microparticles in a food matrix over a period of time. Although this may initially seem alarming as it suggested potential compromise of the protection efficiency of the matrices over time, considering the normal shelf life of yoghurt, which is generally between 20 and 40 days, and viable counts obtained after such period in this study, the results indicated that the encapsulated cells will be sufficiently protected for the duration of yoghurt’s shelf life. Future studies employing sensory analysis with trained panellists are however needed to establish whether prolonged refrigeration of yoghurt containing Vegetal-inulin encapsulated *B*. *longum* LMG 13197 will not negatively affect the texture and taste of yoghurt.

The continued production of acids by bifidobacteria cells possibly contributed to the drop in pH of yoghurt containing unencapsulated cells (Samona et al. 1996). It has been reported that bifidobacteria produce acetic and lactic acids at proportions of 3:2, which when in excess can lead to undesirable effects in yoghurt (Arai et al. 1996). Conversely, Vegetal and Vegetal-inulin matrices minimized alterations to pH of yoghurt by retaining the cells and possibly the metabolites produced by the cells. Thus, the quality of yoghurt was less likely to be negatively affected. Kailasapathy (2006) reported that post acidification was slower in yoghurt samples containing encapsulated probiotics as opposed to those containing free probiotics after 6 weeks of storage at 4 °C. Other studies have reported slight decrease in pH values of yoghurt containing prebiotics over storage time (Akalin et al. 2004; Boeni and Pourahmad 2012).

Colour is a unique characteristic in yoghurt, which if negatively affected, can lead to rejection of yoghurt by the consumers, leading to increased economic losses (Arai et al. 1996). Hence, it is important that the colour of yoghurt after fortification with encapsulated probiotics is monitored to ensure that it remains of the quality acceptable to consumers. The increase in ${\text{L}}^{*}$ and ${\text{b}}^{*}$ values of the samples indicated the tendency towards white and yellow colours (Aryana and McGrew 2007). This might be attributed to the colour of the ingredients present in the yoghurt samples, which include the whitish colour of inulin and the white to cream colour of milk. The affinity to a more yellowish-white (cream) colour was seen with the addition of inulin as reported by Damian (2013), who found a high ${\text{L}}^{*}$ value for yoghurt samples containing inulin. The reduction in ${\text{a}}^{*}$ values might be attributed to the presence of riboflavin, a green compound which has been regarded as part of milk’s natural pigment concentration, and thus contributes to the colour of milk (Nozière et al. 2006). Although all the yoghurts appeared cream in colour at the end of storage, the total colour difference observed between the unencapsulated and encapsulated samples suggests that addition of Vegetal and Vegetal-inulin encapsulated *B*. *longum* to yoghurt were minor to not obvious to the human eye and will have no negative effect on its acceptability by consumers (Martínez-Cervera et al. 2011).

## Conclusions

Freeze dried Vegetal BM 297 ATO microparticles offered protection to *B. longum* LMG13197 cells during exposure to simulated gastrointestinal fluids and in yoghurt. The microparticles protected bacteria from gastric acidity and subsequently released sufficient amounts of viable cells into the simulated intestinal fluid, an indication of release of sufficient cells for colonization of the colon. Post acidification of yoghurt was minimized in the presence of encapsulated *B*. *longum* and its total colour change was considered minor or not noticeable to the human eye. In all cases, Vegetal-inulin microparticles were more protective to *B*. *longum* than Vegetal alone. Therefore, Vegetal-inulin microparticles were suitable for incorporation of probiotic cultures into fermented dairy products such as yoghurt.
